# “*If They Help Us, We Can Help Them*”: First Nations Peoples Identify Intercultural Health Communication Problems and Solutions in Hospital in Northern Australia

**DOI:** 10.1007/s40615-024-02160-4

**Published:** 2024-09-30

**Authors:** Vicki Kerrigan, Stuart Yiwarr McGrath, Rachel Dikul Baker, Jeanette Burrunali, Anna P. Ralph, Rarrtjiwuy Melanie Herdman, Tiana Alley, Emily Armstrong

**Affiliations:** 1 Menzies School of Health Research, Charles Darwin University, PO Box 41096, Casuarina, NT 0811 Australia; 2 Independent Scholar, Darwin, Australia; 3 Bininj Kunwok Regional Language Centre, 5 Gregory Pl, Jabiru, NT 0886 Australia; 4 Department of Medicine, Royal Darwin Hospital, Darwin, NT Australia; 5 Djalkiri Foundation, 11 Arnhem Road, Nhulunbuy, NT 0881 Australia; 6 Northern Institute, Charles Darwin University, Darwin, NT Australia

**Keywords:** Intercultural communication, Cultural safety, First Nations, Healthcare, Codesign, Systemic racism, Interpersonal racism

## Abstract

Effective intercultural communication between First Nations peoples and healthcare providers in colonised countries is required to deliver equitable healthcare and improve patient experiences and health outcomes. This paper presents First Nations peoples’ perspectives and proposed solutions to problematic communication experiences at Royal Darwin Hospital in northern Australia. The study’s methodological foundations comprise decolonising principles rooted in Critical Race Theory, Freirean pedagogy, and cultural safety. Eleven individuals from diverse First Nations backgrounds receiving treatment at the largest hospital in the Northern Territory, participated in in-depth interviews conducted in their preferred languages. Data were inductively analysed, adapted from a constructivist grounded theory approach and guided by First Nations knowledges. First Nations patients at Royal Darwin Hospital described a culturally unsafe hospital due to ineffective intercultural communication. Patient “counterstories” recounted instances of confusion, aggression, healthcare provider resistance to shared decision-making opportunities, pressure to abandon cultural protocols, and institutional neglect. Poor communication incited anger among staff and patients, and contributed to experiences of racism, missed appointments, clinical mistakes, patients prematurely discharging before completing treatment, and patients experiencing financial troubles and homelessness. In a spirit of generosity, patients proposed solutions focused on relationship building and mutual understanding so that new decolonised systems, which draw on First Nations and Western knowledges, can be codesigned. This study serves as a call to action for policymakers, administrators, and healthcare providers to prioritise improving intercultural communication by addressing the behaviour of staff and changing systemically racist policies in the pursuit of health equity and cultural safety for First Nations peoples.

**Supplementary Information:**

The online version contains supplementary material available at 10.1007/s40615-024-02160-4.

First Nations peoples in colonised countries experience significant disparities in healthcare access, quality, and outcomes [1–5]. In the Northern Territory (NT) on the continent colonisers called “Australia”, First Nations peoples make up 25% of the population however approximately 70% of patients at the NT’s largest hospital, Royal Darwin Hospital (RDH), identify as First Nations [6, 7]. Disproportionate rates of ill health are due to the ongoing impact of colonisation. Over the last 50 years, culturally unsafe care in hospitals has resulted in death, absence of informed consent, high rates of self-discharge before treatment is complete, amputations without patient permission, and patients experiencing racism impacting both mental and physical health [8–12]. These unacceptable outcomes are commonly connected to poor patient-provider communication [13–15]. Research has found that when intercultural communication between First Nations peoples and healthcare providers is culturally safe, patient experiences, engagement and outcomes improves and staff burnout is reduced [16–19].

However communication in healthcare, particularly in hospitals, is often dismissed as a “soft skill” in favour of “hard” clinical medicine which centres the biosciences as foundational to healthcare [20]. This Western approach is not conducive to healing for many First Nations peoples worldwide [21]. This is because for First Nations peoples good health is a holistic concept that includes relationships with family and community; culture and identity; self-determination; education, work and responsibilities; connection to country and physical and mental health [22, 23]. If healthcare providers wish to provide an equitable service to First Nations peoples, it is imperative “counterstories” regarding what constitutes good health from patients are heard and perspectives are incorporated into healthcare delivery [24].

While intercultural communication in Australian hospitals can be improved by working with Aboriginal Liaison Officers and language interpreters and the employment of more First Nations health professionals, tackling race related inequities should not be the sole domain of those who experience inequities [11, 25, 26]. Statistically it is impractical to expect First Nations health professionals, who constitute about 1.2% of the health workforce nationally, to assume responsibility for addressing systemic problems [27]. Additionally, First Nations health professionals leave the workforce after experiencing burn out which has been attributed to experiences of racism and trauma [28]. To allow White Australians to reassign to First Nations peoples “the work White people need to do on themselves” [29 p.357] perpetuates “othering” and potentially enlarges the disparities that already exist. As Freire [30] noted, to create an equitable society, the descendants of the colonisers and the descendants of the colonised have different tasks to complete but this should be done in dialogue with each other.

This paper documents First Nations peoples’ experiences of culturally safe intercultural communication at RDH. It is imperative to document patient stories because cultural safety, which is about the “analysis of power and not the customs and habits of anybody”, can only be determined by patients [31 p.181]. Patient stories which counter the dominant racist narrative and expose new ways of thinking have transformative potential [32–34]. Personal stories have the power to shift hearts and heads and eventually can inform the development of evidence-based policies and interventions that can improve quality and safety and healthcare access and outcomes [35, 36]. Critical Race Theorists argue that “counterstories” from marginalised peoples are ‘the cure’ to ongoing racial inequities [24].

Two broad research questions were explored: (1) Considering cultural safety principles, how do First Nations peoples at Royal Darwin Hospital perceive and experience intercultural communication with healthcare providers, and (2) What solutions do they propose to address problematic communication practices to ensure culturally safe and equitable healthcare delivery? Therefore, the aim of this paper is twofold. Firstly to present RDH patient experiences of intercultural communication and proposed patient solutions and secondly to contribute to the evidence that communication in healthcare should be “acknowledged as a significant determinant of health outcomes” [19 p.560].

## Methods

### Study Design

This qualitative study was nested in a larger Participatory Action Research (PAR) [37] project called the Communicate Study Partnership [38]. The project aim was to improve First Nations peoples’ experience of hospitals in the NT by exploring and implementing culturally safe communication practices. The research was underpinned by decolonising principles rooted in Critical Race Theory (CRT), Freirean pedagogy, and cultural safety [30, 31, 39, 40]. These philosophies shaped the study design, research aims and methods including the creation of a culturally and racially diverse research team, the preference to conduct interviews in First Nations languages and the exploration of participant “counterstories”. Our work was also guided by First Nations knowledges and approaches to research which recognise the value of reciprocal relationships which also aligns with PAR praxis [37]. Our analysis drew on aspects of constructivist grounded theory [41, 42]. This methodologically rigorous framework allows for the emergence of nuanced insights that are deeply rooted in the lived experiences of the participants who may have been previously silenced by hegemonic research practices. Our work takes a critical approach towards hegemony and aims to actively challenge the dominance of Whiteness in research and in healthcare. Approval to conduct the research was granted by the Human Research Ethics Committee of the Northern Territory Department of Health and Menzies School of Health Research (HREC-22-4297) and the Research Governance Office, Northern Territory Department of Health (EFILE2022/13836). 

### Researcher Backgrounds

VK is a White intercultural health communication researcher and practitioner. SYM is a Gumatj man from the Yolŋu nation; Djambarrpuyŋu, a dialect of Yolŋu Matha, is his first language. He is an Aboriginal Health Practitioner and researcher. RDB is a Yolŋu woman from Ŋurruyurrtjurr from the Djambarrbuyŋu clan; Dhuwal is her language. She is a cultural facilitator, interpreter, and accomplished weaver who is undertaking a Bachelor of Arts in Anthropology. TA is an Alawa and Marra woman from Darwin NT starting out her professional life a research assistant at Menzies School of Health Research. RMH is a bilingual Gälpu women from the Yolŋu nation. She is the Manapanami (Chief Executive Officer) of the Djalkiri Foundation, chair of the Miwatj Health Aboriginal Corporation and a researcher. JB is a Kunwinjku speaking Djalama women. She is a language worker at Bininj Kunwok Regional Language Centre (BKRLC) and an interpreter at the NT Aboriginal Interpreter Service. APR is a White global health researcher and infectious disease clinician. EA is a White Australian collaborative intercultural communication researcher and a speech pathologist. Some First Nations researchers on our team have had personal lived experiences of the same issues reported in this paper.

### Study Setting

Research was conducted on the unceded lands of the Larrakia people known as Darwin, NT. RDH is a 360-bed facility managed by the NT government. Approximately 70% of patients identify as First Nations and only 7.8% of NT health staff identify as First Nations (the report does not provide a breakdown of staff diversity at RDH) [43]. Many RDH staff, from southern Australian states or overseas, experience culture shock on arrival in the NT [17]. Most are unfamiliar with the diversity and strength of First Nations cultures, arrive with or readily develop negative stereotypes and bias shaped by the media or superficial observation, and are ill-equipped to provide culturally safe care [44, 45].

### Participant Sampling

A purposeful sampling strategy [46] identified key informants who could share personal experiences regarding intercultural communication at RDH and the changes required to improve experiences of culturally safe care. Researchers aimed to include participants from different lands, clans, and language groups, who had experienced healthcare in a range of hospital services including: surgical care, renal care, allied health services, and parents of children receiving care in paediatric wards. First Nations patients and family members were eligible to participate if they were over the age of 18 years, had experience as an RDH in-patient, and able to consent to participate. Participants were invited through personal and professional networks of the authors. After patients indicated interest in the project, the project information sheet and consent form were discussed in the patient’s preferred language before the patient signed the consent form.

### Data Collection

Face-to-face semi-structured interviews were conducted with patients and discussion was centred on what patients wanted to share regarding their experiences of culturally safe communication. Interviews were conducted at a location chosen by the patient; a private room at RDH; bedside on the ward; in a seating area outside the hospital; the researcher’s office; or at hostel accommodation. All interviews were audio recorded except for one participant who declined to be recorded but allowed researchers to take notes. Patients were thanked for their participation with a supermarket voucher. Researchers spoke Yolŋu Matha, Kunwinkju, and English which meant Yolŋu Matha and Kunwinkju speaking patients could record their stories in their first language. Researchers led the data collection process with culturally specific and safe conversational approaches. Additionally, through the moral bonds of kinship Yolŋu and Bininj researchers had relationships of accountability with patients from their Nations thereby ensuring data was collected in a culturally safe manner [47].

### Data Analysis

Drawing on constructivist grounded theory, data were inductively analysed and guided by First Nations knowledges and decolonising theories. Interviews recorded in Yolŋu Matha and Kunwinkju were translated into English by SYM, RDB, and JB. During translation, SYM, RDB, and JB provided contextual explanations, which were documented as analytical memos on transcripts [41]. These explanations, sometimes referred to as “cultural intuition” [48], provided insights into Yolŋu and Bininj worldviews regarding communication norms. Interviews recorded in English were transcribed verbatim. A second round of analysis was undertaken by SYM and VK who uploaded transcripts into NVIVO12 to code similarities, differences, and patterns in the data. If the interview was recorded in English, VK and SYM coded interviews while simultaneously listening to the audio and reading the transcript. Interviews in Yolŋu Matha were listened to a second time by SYM. The process of listening again to the audio minimised dematerialising voices to words disconnected from lived experience [49]. The combined analysis led to categories that described shared and divergent experiences. Categories were further refined through discussions with co-authors. Consistent with Critical Race Theory, this process revealed “counterstories” [24] which formed the basis of the findings. Some participants chose to use their real name in the publication thereby ensuring sovereignty of perspectives, others requested pseudonyms.

### Terminology, Language, and Ethical Considerations

Regarding terminology, First Nations peoples are identified in relation to their Nation and/or language group. When necessary, we use ‘First Nations’ which recognises the diversity of nations who hold unceded sovereignty over the continent known as Australia. In this paper, the term Yolŋu has two meanings: it can refer to people who belong to the Yolŋu Nation and to people who belong to other First Nations. Balanda is a Yolŋu word used to refer to non-Indigenous people or White people. Non-Indigenous researchers capitalise the word White to associate themselves with the socially constructed racial category defined in Whiteness studies [50]. The term White refers to people who, knowingly or unknowingly, benefit from a racialized society [51]. Regarding language, we have included Yolŋu words which relate to intellectual academic concepts that cannot easily be translated into English. These Yolŋu phrases have been included because they reflect the essence of Yolŋu philosophy and highlight the complexity involved with intercultural communication. In this way, we overturn the idea that Western knowledge is the source of “higher orders of thinking” [40 p.51].

## Findings

Between 5th October 2022 and the 20th January 2023, 11 people were interviewed. Stories were shared by: Yolŋu patients Dorothy, Anita, Jeffrey, Michael, and mother Baŋaḏitjan who was with her hospitalised 14-year-old child; Bininj patients Dell and John plus his wife Margaret; Yammirr woman Moana; Torres Strait Islander mother Charlene who was with her 6-month-old child; and mum Rebecca, who was with her 16 month old child, who identified as First Nations but did not share her Nation or clan.

Patients reported feeling unsafe due to interactions with healthcare providers, citing instances of miscommunication, aggression, and a lack of attentive listening which limited shared decision-making opportunities. Poor communication led to patients self-discharging, clinical mistakes, missed appointments, and financial difficulties for patients. Differing worldviews between Western and First Nations communication norms created further challenges. Communication issues exacerbated the systemic and interpersonal racism experienced by First Nations patients, leading to decisions made without proper consultation or informed consent, and causing psychological and physical distress. To improve intercultural communication between providers and First Nations peoples, patients emphasised the need for healthcare delivery to be codesigned, recognizing and respecting diverse cultural perspectives to build trust and understanding between providers and patients. Findings summarised above will be explored in detail below.

### Intercultural Communication Failures and Institutional Neglect: “Sometimes They Are Very Rude”

Patients felt scared, disrespected, belittled, uncomfortable and consequently angered by interactions with healthcare providers. Michael explained that the hospital was not designed for First Nations peoples: “The hospital is full balanda …the hospital is for white people, it’s theirs”. Michael was anxious and distrustful of healthcare providers when he was admitted to hospital:

When I came here the first time, I was thinking through a lot of scenarios in my mind like, ‘Are these people going to slowly attack me with different medications, to the point where I die?’

Dell said she often felt “out of place”. She observed examples of miscommunication between patients and providers which she described as “really scary”.

Patients cited instances of feeling ignored and said staff with bad attitudes spoke in an aggressive manner. Michael said: “when we speak, they don’t listen to us”. Moana, who previously worked as a nurse at RDH, said staff priorities were “twisted”. She said:

These mob they’re always rushing, rushing…You know, instead of listening or seeing their patient, they are always doing their own thing, paperwork and all that….Sometimes they just come in, and just quick talk and walk out. Like as a patient, I want to say something to that doctor and ask more questions about the medication. They just get up and walk away. They’re very rude.

Dorothy said staff should speak to patients “gently”, so they feel “warmth” because many people have been forced to leave their families to receive hospital treatment. Anita relocated from her community 450 kms east of Darwin to receive dialysis treatment at RDH. She is happy with the clinical treatment, but feels patronised by staff who treat her like a “child”. However, Jeffrey a dialysis patient for over 10 years, believed healthcare providers “have to be bossy. Sometimes you have to wait for the doctors or nurses….They come in their own time. You can’t push them”.

Communication also failed because of the medical language used and pace of delivery which contributed to patients feeling overwhelmed and confused. Patients said communication was further complicated when interacting with healthcare staff who spoke with a non-Australian accent. Jeffrey suggested staff should slow down, give the patient time to ask questions, “use small language. Plain. Not complicated words.…If you talk too much, I’ll get confused”.

Patients also described communication issues outside the hospital wards which contributed to feeling unsafe. Rebecca, John, and Dell, all from remote communities, relied on the hospital bus service to get to appointments. They described the bus drivers as “rough”. In this context, the word rough means rude. Dell said the bus drivers “don’t respect us, the sick people”. John, who used a walking frame to help with mobility, told a story in which the bus driver told him to hurry up:

That bus driver was rough. He was saying ‘Come on hurry up, hurry up!’. I turned around and said to the bus driver – ‘Why are you getting rough to me? Can you calm down or go slowly’. Don’t be rough to the patients…..Tell him (the bus driver) not to do that anymore, to be rough to the patients. They’re working for us patients.

### Consequences of Culturally Unsafe Communication: “If We Feel No Good, We Disappear”

Firstly, patients self-discharge before treatment is complete. Michael said when Yolŋu feel disrespected or scared, they will exert the limited power they have by leaving the hospital without talking to the providers:

If they hurt our feelings, we will just walk away, leave…..If we feel no good, we disappear secretly. ‘Cause I’ve seen some of the patients they walk out, jump in the taxi and just disappear. There is probably something wrong here in the wards. The white people didn’t take care of them.

Poor communication contributed to clinical mistakes. Mum Charlene told a story about a “very stressful night” in the RDH Emergency Department with her 6-month-old baby. After waiting for 5 hours, the family was seen by a doctor who Charlene described as “a good bloke…just trying to do his job” but lacking the skills for the task at hand. Having observed healthcare providers caring for her baby over several months, Charlene had developed a comprehensive understanding of the procedure required. Concerned for her baby’s welfare and wanting to help the doctor, Charlene attempted to show the doctor a video she had recorded on her phone of another healthcare provider performing the treatment required: “we were just trying to help him, we could see he was stressing”. However, her attempt to contribute to the care of her baby was ignored by the doctor. Charlene said doctors then used the wrong equipment and burnt her baby’s leg. Asked why she thinks the doctor didn’t listen, Charlene said: “I’m just a mother”. Charlene believes providers should engage with families who want to be involved with the care of their loved ones:

Take in what the mother says, first of all….I’ve been coming in here every week, it’s like a second home….and they still didn’t listen to me. So that felt very, that made me feel like shit, a lot.

Inappropriate communication led to missed appointments and transport issues. When patients are discharged from hospital, they are handed written information which is not explained in Plain English. Dell said: “they miss their doctor’s appointment and that’s it. I say to them: ‘What happened?’ They say: ‘I don’t know.’ They were given a letter to read”. Dell explained the written discharge communications caused financial problems for patients: “they miss their flight and they miss their bus so they have to pay more. But they got no money to pay”. This led to patients being stranded and homeless in Darwin unable to return to their communities.

Ineffective communication contributed to a lack of trust between patients and providers. Patients including John said that some doctors “hide the truth”. John said: “Sometimes they don’t give us the full story, they give us a short story, a light explanation”. Dorothy said providers should educate patients on their conditions and the reasons behind treatment plans so they can play an active role in their care: “They have to tell us, so that we as Yolŋu can learn about it”. Baŋaḏitjan explained that when the clinical condition is not well explained, that can lead to confusion and the patient thinks “the doctors are lying”. Anita said the way a healthcare provider can overcome the perception of lying is to develop a relationship with the patient.

### Providers Need to Change How They Communicate: “Your Thinking Is Different to Mine”

Patients explained that communication issues exist because Western communication practices dominate the hospital. Yolŋu patients Michael and Dorothy said there is an expectation that patients should adjust how they communicate to accommodate healthcare providers but healthcare providers are rarely expected to adapt their communication styles to accommodate First Nations patients. Dorothy explained mainstream laws don’t recognise Yolŋu laws and this contributes to communication problems: “We don’t recognise each other. Our laws are still not recognising each other”. During time as an inpatient, Dorothy cautioned providers against imposing their own thinking onto her:

I told them, ‘Your thinking is different to mine. You’re not communicating well, you have to come to me first and sit down and find out who I am and see what you can learn about me’. And I told them, ‘It’s not just about you, where’s my path? How do I communicate as a Yolŋu person?’.

Patients said treatment plans were created by non-Indigenous healthcare providers through an ethnocentric approach shaped by Western worldviews. Michael said: “They expect me to transfer my thinking to their thinking, they want me to think like them”.

### Systemic and Interpersonal Racism: “They Should Respect Us”

When communication is not culturally safe, cultural protocols are ignored by the systemically racist system. For example, patients felt pressured to make decisions quickly without consulting family as healthcare providers prioritised hospital timelines over cultural protocols. Michael said: “They don’t understand the definition of patience, they only think of themselves and they are just forcing people”. For most First Nations peoples, decisions are made with family, not by individuals. Baŋaḏitjan had three children with rheumatic heart disease. As the mother, she made decisions in consultation with her Elders. The same protocol applied to Elder Dorothy:

I must talk with my family, and I have to consider my kinship responsibilities. And they (family) must know the story about what’s happening with me. We have a process for this. Just like the white people, they look to their family to discuss but sometimes they don’t; they make decisions themselves because they have a different process in their culture. And in ours there is a rope*, a rope that connects us called Gurruṯu and it extends further, we can pull that rope and gather everyone so that everyone knows what’s going on. Our culture tells us that.

(* “rope” is a metaphor. Rope refers to a “family tree” which may comprise genetic or kinship connections)

Another example of systemic racism was the hospital complaint processes. All patients who were asked if they knew how to lodge a formal complaint were unaware of the process. Dell said some nurses yell at her: “So, I get aggressive too to them. ‘Don’t you dare scream at me like that.’ You know, I say that back to them. ‘Otherwise I will make a complaint about you’”. Asked if she had ever made an official complaint, Dell said no. Margaret, wife of John, said that if they wanted to make a complaint about RDH they would contact an unrelated non-government organisation. Moana said people don’t complain because they fear retribution from healthcare providers and because complaints must be lodged in written English which may not be the patient’s preferred language. Despite dissatisfaction with her care, Moana has never lodged a complaint because she prioritises supporting her children and grandchildren: “There’s no time to sit and write a formal complaint when you got other things going on”.

Dell is a former Aboriginal Health Worker and an interpreter; she has been a renal patient at RDH for over 10 years. She said: “I think it’s a racist place”. As an example, Dell shared her reflections on anti-racism posters which were prominently placed around the hospital including at the reception of the Aboriginal Liaison Office. The posters read: “Respect those who care for you. We say no to all forms of violence, threats, swearing and racism”. After seeing the posters which urged patients and visitors to respect staff, Dell said there should also be posters that tell staff to respect patients: “Why don’t they respect us sick people? You write that down. They should respect us”.

When asked about interpersonal experiences of racism in healthcare some patients said they were treated differently because of their racial background. Patients observed that White people were treated with more respect and given more time; Michael said that “if a white person is sleeping, they are respected and left alone”. Dell and Moana felt doctors and nurses did not want to touch them. Emphasising how culturally unsafe she felt Moana explained that if any of her 33 grandkids required hospital care she would “take them somewhere else” for treatment. One patient Jeffrey did not relate to the concept of interpersonal racism: “I don’t see racism much but maybe I don’t understand it”.

Patients also spoke about a heightened awareness of nonverbal communication and feeling bad vibes from providers. Michael explained that Yolŋu are attuned to subtle cues such as tone of voice and the physical way tasks are completed: “I see their attitude, how they act, even if they put something on the table aggressively”. He suggested that balanda (White people) are not as skilled as Yolŋu at noticing nonverbal cues:

‘Cause we Yolŋu, we know. We can feel bad stuff. How that person is behaving, ‘cause we are Yolŋu and that person is white. That balanda won’t feel our vibe but we Yolŋu, we can see their vibe accurately. We can feel it when they are talking, that they are holding something sinister, it’s problematic inside them, a negative emotion.

### Developing Trust: “When the Health Professional Treats Me Well Then I Will Trust Them”

Patients said communication would improve if they could trust the providers. John said some providers were good and some were bad, he said they must earn his trust: “When the health professional treats me well then I will trust them”. Anita, a dialysis patient at RDH for over 3 years, said there are some providers whom she trusts and consequently, she teaches them words in her language which strengthens mutual understanding and respect.

Dorothy spoke about ceremonial leaders, holders of law/lore, and professors in Yolŋu culture whose authority and status is not recognised by the hospital systems. Dorothy would like to see the power imbalance rectified: “This is why doctors should recognise us because we have power with us and (they have power) with them”. Michael said, in addition to providers learning about their patients’ cultures, providers should also share about themselves so the patient can get to know the professional on a personal level. Patients valued time with providers willing to build relationships because it opened opportunities for conversations about how patients and families can manage their health conditions. Charlene who spoke about the stressful experience in the Emergency Department with doctors she had no relationship with attending to her child’s procedure (explained above) also had a positive experience with a physiotherapist. Charlene said the physiotherapist operated according to “the patient’s timing” as opposed to hospital schedules. She appreciated this patient-centred approach because the physiotherapist invested time explaining her son’s condition and she enjoyed developing a personal connection which fostered trust:

Just normal having yarns throughout the process. It’s just like, that little connection there. Like you can talk to them about I don’t know, anything, life (laughs). Or just with baby. (They will ask), ‘What’s going on with baby? How is he?’ They teach me a lot about his (condition). I feel like I’m a physio myself sometimes…We come in every week, every Wednesday. This is like his second home.

### Codesigning a New Way of Working: “Create the Solutions Together”

Patients said improving communication required urgent attention due to the increasing and disproportionate rates of illness experienced by First Nations peoples. Patients were confident communication could be improved by changing systems to better support the patient’s social and cultural needs. Yolŋu Elder Dorothy said healthcare delivery should be codesigned because currently First Nations worldviews are not recognised by the “foreign” system:

If they help us, we can help them. Create the solutions together. But they have to do the work first, and open pathways for us because that place [the hospital] is full of complicated foreign things and a lot of different systems.

The foreign systems created challenges for patients. Mum Rebecca travelled from her remote community 730 kms southwest of Darwin to RDH with her 16-month-old son, who required surgery. On the day of the surgery, Rebecca and her son travelled by hospital bus from the hostel accommodation to RDH but because the bus was behind schedule the family missed their pre-admission appointment. When Rebecca and her son arrived at RDH, she was told the surgery had been cancelled which caused her significant psychological distress. Fortunately, Rebecca was supported by a nurse who was working in one of three new roles created by the RDH paediatrics department to support families navigate the health system (travel arrangements, accommodation issues, clinical appointments etc.). With support from the paediatric nurse, who understood the “foreign” system, the surgery was rescheduled.

Dorothy appreciated the scientific knowledge healthcare providers possess but said that patient experiences of healthcare would improve if providers recognised that patients also have knowledge which can support their wellbeing.

Because that’s how we can understand each other: generosity and voice…. Recognise each other, agree with each other, and disagree with each other together. From their perspective and from our perspective.

Dorothy and researcher SYM spoke about the Yolŋu concept of Galimiṉḏerrk which is a process of freshwater and saltwater mixing together. This concept can be used as a metaphor for two cultures or systems interacting. They agreed that if the hospital culture could merge with Yolŋu cultures, then Dorothy said “inside we can turn the system around, and that’s when we will recognise each other” in a new equitable way:

Embedding our culture into their culture has never happened before. This is rorru (a sacred process of great importance) which establishes dhanuyuman (a gold standard). I am suggesting a new pathway today so they can see. From there we can be together on the same level. Indigenous and non-Indigenous.

To illustrate her point about Galimiṉḏerrk which can create a new gold standard for practice, Dorothy told a story about how she built a connection with doctors and nurses before she underwent surgery. Lying on the hospital bed in the surgical ward Dorothy felt stressed and was concerned that doctors and nurses were not communicating well so she asked them to stop and pray with her.

I said, ‘Gather around. You the doctor and those people who are surgical specialists, make your communications better. You are thinking differently and the surgeons are thinking differently.’ And honestly I was seeing that. I told them, ‘You have to communicate with each other first, before you talk to me’.…. That’s what I said. ‘Now, I’m going to pray for us. Hold each other’s hands.’ Those bosses that do surgery, I told them to hold each other’s hands and pray. And then we prayed.

Following the prayer, Dorothy said the team decided to play gospel songs from Yolŋu singer Gurrumul. For Dorothy, this demonstrated a newfound awareness among the medical team who sought to create a more comforting environment in which both cultures were valued:

They listened to me. That’s it. And then after that, they felt it and they saw, and they understood that they needed to understand each other first before they can understand me. And that’s why they decided to play Gurrumul singing gospel songs next to me.

## Discussion

The experiences shared by Yolŋu, Bininj, Yammirr, Torres Strait Islander, and other First Nations peoples, who were patients at RDH over 5 months during 2022–2023, illustrate the impact of culturally unsafe intercultural communication. Patient stories detailed instances of miscommunication, aggression, healthcare provider resistance to shared decision making opportunities, pressure to abandon cultural protocols, and institutional neglect. The consequences of culturally unsafe communication included angry staff and patients, missed appointments, gratuitous concurrence, clinical mistakes, transport problems, and patients self-discharging before completing treatment. Ineffective communication also contributed to patients experiencing financial difficulties and homelessness perpetuating a cycle of disenfranchisement from healthcare, employment, and education opportunities.

While most healthcare providers self-identify as humanitarian anti-racists who are beyond “crass prejudicial views about minority groups” [52 p.1359], First Nations patients at RDH revealed daily experiences of interpersonal and systemic racism in the clinical and non-clinical space. Medicine has a long history of racism specifically in northern Australia, at the start of the twentieth century, when medicine promoted scientific racism and fostered a “racist nationalist culture” [53 p.256]. The Australian Commission on Safety and Quality in Health Care recognises that health services have been places of violence:

The health system in the past included segregated wards and service entrances, deliberately different (substandard) care, forced removal of newborn babies from mothers who were considered ‘not competent’ or not able to provide the ‘right upbringing’, and removal of children from home while parents were sick in hospital and failure to return these children to their parents’ care. [54 p.22]

While these policies and practices are in the past, they are within living memory and First Nations peoples requiring hospitalisation continue to experience inequitable healthcare. Racism today often involves small, seemingly mundane brutalities referred to as microaggressions [5, 55]. Patients at RDH spoke about experiences of racism which included disrespect such as ignoring patient questions and rude bus drivers; harassment included yelling at the patient and disturbing sleep to conduct healthcare activities; devaluation was evidenced by rejecting parents’ attempts to contribute to their child’s care; and instances of dehumanisation included expecting patients to behave and communicate according to Western norms and excluding family from decision making [56, 57]. Instances of colour-blind racism and White ignorance were evidenced by the culturally inappropriate discharge procedures and complaint processes [52]. One patient denied the presence of racism but also said he did not have a comprehensive understanding of the concept. It is important to unpack this comment which could be misinterpreted as indicating that racism does not occur at RDH. Firstly, Yolŋu researchers SYM and RMH explained that Yolŋu may find it difficult to talk about race and racism because battling for power is something Yolŋu did not do before invasion. Secondly, racism can be an ordinary part of daily life therefore it is unidentifiable and unremarkable [58]. For others, discussing experiences of racism can be retraumatising [59]. In Australia, “racial literacy is dismally low and talk of race and racism is silenced” [60 p.2]. The denial of racism favours colonisers as it works to uphold Whiteness as a structure of oppression [60 p.2]. Researchers and policymakers must develop a nuanced understanding regarding the ways in which racism manifests because patient experiences of discrimination by healthcare providers remain a significant barrier to health [1, 59, 61–63].

Solving issues identified requires a long-term commitment to systemic change. The patients' perception that the hospital is “foreign” emphasised the need to codesign a new decolonised system. To decolonise patient-provider interactions and the larger health system, respectful relationships in which two-way dialogue occurs is necessary [19]. We found when relationships were created between patient and provider, such as the positive experiences shared by patients who developed trusting relationships with a paediatric nurse and a physiotherapist, intercultural communication became safe. Our findings are backed by research which found when providers and patients have a “social relationship” the delivery of clinical care and patient experiences improve [64]. To build the social relationship, patients wanted healthcare providers to learn about their cultures and they also wanted to find out about the healthcare provider personally. This assists patients to explore “the vibe” of the provider which is characteristic of high-context cultures [65]. In high-context cultures, high value is placed on contextual factors in interpreting a communication; individuals prioritise relationships of reciprocity, and communication tends to be subtle and value is placed on nonverbal communications [65], whereas in low-context cultures, communication is explicit and individualised and there is little requirement for relationships [65]. Generally, low-context cultures align with Western communication norms which promote the connection of people through acts associated with capitalism; value is placed on what people do to make money and ownership of goods not who they are in terms of family and culture [40, 66]. Low-context cultures and associated communication norms dominate hospitals in Australia.

A common theme that ran through all patient stories was the lack of recognition of First Nations policies and the imposition of Western norms. For the patients, communication occurred at the “cultural interface” which is a contested space where “things are not clearly black or white, Indigenous or Western” [67 p.9]. RDH patients expressed a spirit of generosity and a desire to connect at an individual and institutional level which could lead to the creation of new pathways or systems [68].

Yolŋu patients spoke about the importance of healthcare providers and systems recognising Yolŋu concepts, policies, and laws such as galimiṉḏerrk. Galimiṉḏerrk describes the process of salt and fresh water coming together to create something new [69]. In the beginning, when salt and fresh water bind there is turbulence but eventually it becomes calm when an equilibrium is found. In this way galimiṉḏerrk is akin to decolonisation. Decolonisation does not require a total rejection of Western knowledges. Rather, like galimiṉḏerrk, it involves disorder and chaos as First Nations knowledges and Western knowledges meet to create something new where power is shared [40]. Galimiṉḏerrk is a natural process of creation in which things that are different can merge and become stronger; “When galimiṉḏerrk occurs there is no power imbalance” [69]. Yolŋu patients also explained the importance of relationships with the concept of “gurruṯu”. Gurruṯu explains how people are connected through kinship, how to communicate and how to behave when in reciprocal relationships [70]. Herdman and Rafferty [71] argue that to create decolonised culturally safe professional spaces gurruṯu must be foundational.

By centering gurruṯu in the workplace, Yolŋu knowledge systems are validated, and a more human-centred collaborative model of service delivery is possible. While the term ‘both ways’ is often used to describe collaborative work practices, we argue that this concept is at best misunderstood by governments, departments and individuals and, at worst, sustains dominant ideology. For a model or approach to be considered ‘both ways’ the validation of knowledge systems and language is needed, Yolŋu governance must be prioritised and a critique of power is necessary. When we practice both ways as a theoretical and applied framework, relational ways of doing ‘business’ is at the heart of our approach. Deep collaborative engagement, critical reflection and a balance of power is critical in intercultural spaces - this is gurruṯu [71].

When gurruṯu is not recognised or practiced, miscommunication can occur. In practice, “this can negatively impact the medication or treatment plan for the patient” (RDB memo, 12th February 2023). At an institutional level, without relationships, new systems cannot be created.

To address intercultural communication issues identified, a significant shift in the attitudes, knowledge, and skills of providers is required alongside the establishment of accountabilities for health organisations through quality improvement strategies [61]. We identified four areas for quality improvement. Firstly, our findings support calls for ongoing training programmes for healthcare providers that focus on intercultural communication skills and anti-racism praxis [1, 72–74]. During undergraduate training, doctors learn how to control communication to solicit biomedical information. Students “enter medical school with better communication skills than when they leave” [75 p.22]. This learnt communication style diminishes trust between patients and providers, and patients lose confidence in healthcare [73]. By changing how providers speak with First Nations peoples, “we can alter the power dynamics and cultural safety of health consultations” [13 p.110]. Training programmes, which support providers to develop their critical consciousness and provide practical information on how to adapt communication styles using Plain English, have been successful in shifting negative attitudes and positively changing practice [32–34, 76].

Secondly, efforts should be made to improve the delivery of discharge information. Information is commonly shared in verbal and written forms but if the communication was not in the patient’s language, or the verbal communication was tailored to a low-context communication style, then effective information transfer may not have occurred. From the hospital’s perspective, ineffective communication places extra pressure on staff and the overburdened health system when the patient may be readmitted for the same condition [11]. From the patient’s perspective, missing follow-up appointments or not taking prescribed medication impacts health outcomes, family life, and the ability to contribute to social, educational, economic, and cultural activities that support cultural continuity [77]. Victim blaming and deficit discourse regarding patients who “do not respond positively to prescribed health interventions” results in experiences of interpersonal and systemic racism [21, 78].

Thirdly, consent processes must be revised to ensure First Nations protocols are adhered to. This could also limit the possibility of clinical mistakes. Previous research has found patients sign consent forms without understanding what they are consenting to, and in some cases, this has resulted in amputations without the patient’s permission [10, 79]. Researcher RDB suggests consent forms should include a tick box which prompts staff to engage family because currently, “The balanda process is taking the family’s responsibility away from them. Taking responsibility away is the same as taking power away from people, i.e., this is a racist act*”* (RDB memo, 12th February 2023).

Finally, the mechanisms in place for patients to make complaints must be reviewed. A report commissioned by NT Health found that “Indigenous people are grossly underrepresented in complaints figures” and recommended various mechanisms, such as training Aboriginal Liaison Officers on complaint handling processes, to address this [80]. Experiences at RDH are replicated in other colonised countries. Māori and First Nations peoples of Canada are also reluctant to complain due to a sense of powerlessness and fear of reprisal from hospital staff [21]. Accessible and culturally safe processes, which allow for patients to lodge complaints in their first language, would empower patients to confidently voice concerns which would allow the system to better respond to patient needs.

## Strengths and Limitations

A methodological strength of the study was that most patient interviews were conducted in patients’ first languages by First Nations researchers. This allowed patients to speak freely about their experiences which resulted in new information about what matters to them [81]. We acknowledge that the patient sample does not represent all First Nations peoples’ perspectives receiving healthcare at RDH; however, hospitals can improve the quality of care by exploring the patient’s insider perspective revealed through key informants [82]. Finally, we recognise that to develop a comprehensive understanding of intercultural communication experiences at RDH, the healthcare provider perspective is also required because intercultural communication occurs between cultures: this perspective has been canvassed in other publications [44, 45, 76, 79].

## Conclusion

First Nations peoples’ experiences of intercultural miscommunication, culturally unsafe practices, and systemic and interpersonal racism at RDH highlight the critical and urgent need for transformative changes in healthcare delivery. In a spirit of generosity and with a desire to connect, patients provided astute and thoughtful strategies for decolonising and co-designing new systems which draw on the strengths of both First Nations and Western knowledges. The patient perspectives underscore the importance of respectful relationships, bidirectional communication, and a commitment to ongoing education regarding culturally safe intercultural communication practices among healthcare providers. Participants also called for recognition and respect for First Nations laws, systems, and expertise. Crucially, successful implementation of the recommendations will require ongoing collaboration between First Nations and non-Indigenous partners. Codesign with First Nations experts must be prioritised for quality improvement in the four areas we have highlighted, i.e., in healthcare provider training and processes for discharge, consent, and complaints.

By recognising the significance of cultural safety and effective, respectful intercultural communication this study serves as a call to action for policymakers, healthcare administrators, and providers to prioritise improving intercultural communication by working with patients and First Nations leaders to create solutions together. Our study provides evidence that culturally safe intercultural communication is preventative medicine.

## Supplementary Information

Below is the link to the electronic supplementary material.

Supplementary file1 (DOC 147 KB)

## Data Availability

Data collected and analysed during the study are not publicly available due privacy issues and ethical considerations. Data may be available from the corresponding author on reasonable request.

## References

[1] Dudgeon P, Bray A, Walker R. Mitigating the impacts of racism on Indigenous wellbeing through human rights, legislative and health policy reform. Med J Aust. 2023;218(5):203–5. 36871199 10.5694/mja2.51862 PMC10953442

[2] Selvarajah S, et al. Racism, xenophobia, and discrimination: mapping pathways to health outcomes. The Lancet. 2022;400(10368):2109–24. 10.1016/S0140-6736(22)02484-9 36502849

[3] Hamed S, et al. Racism in healthcare: a scoping review. BMC Public Health. 2022;22:988. 10.1186/s12889-022-13122-y. 35578322 10.1186/s12889-022-13122-y PMC9112453

[4] Bond C, Singh D, Kajlich H. Canada-Australia indigenous health and wellness racism working group, Discussion Paper Series. Melbourne: The Lowitja Institute; 2019.

[5] Kidd J, Came H, McCreanor T. Using vignettes about racism from health practice in Aotearoa to generate anti-racism interventions. Health Soc Care Community. 2022;30(6):e4020–7. 10.1111/hsc.13795. 35302269 10.1111/hsc.13795 PMC10078765

[6] Australian Bureau of Statistics. Northern Territory: aboriginal and torres strait islander population summary. 2022 [cited 2022 25th October]; Available from: https://www.abs.gov.au/articles/northern-territory-aboriginal-and-torres-strait-islander-population-summary.

[7] Northern Territory Government, NT Aboriginal Health Plan 2021–2031. 2021, NT Health: Casuarina, NT, Australia. Available: https://health.nt.gov.au/__data/assets/pdf_file/0015/1035501/nt-aboriginal-health-plan-2021-2031.pdf.

[8] NT Coroner Court, Inquest into the death of Henry George Wilson aka Albert George Wilson [2018] NTLC 022. Findings of Judge Greg Cavanagh. 2018: Darwin, Northern Territory. p. 24.

[9] Allam L. Darwin hospital left Aboriginal man to die alone and in agony from surgical injury. Northern Territory coroner issues scathing report detailing multiple failures by Top End health service, in The Guardian; 2018.

[10] Brennan G. The need for interpreting and translation services for australian aboriginals, with special reference to the northern territory: appendices. Research Section, Dept. of Aboriginal Affairs, 1979.

[11] Kerrigan V, et al. From “stuck” to satisfied: Aboriginal people’s experience of culturally safe care with interpreters in a Northern Territory hospital. BMC Health Serv Res. 2021;21(1):548. 34088326 10.1186/s12913-021-06564-4 PMC8178868

[12] Askew DA, et al. “I’m outta here!”: a qualitative investigation into why Aboriginal and non-Aboriginal people self-discharge from hospital. BMC Health Serv Res. 2021;21(1):907. 34479571 10.1186/s12913-021-06880-9 PMC8414851

[13] Jennings W, Bond C, Hill PS. The power of talk and power in talk: a systematic review of Indigenous narratives of culturally safe healthcare communication. Aust J Prim Health. 2018;24:109–15. 29490869 10.1071/PY17082

[14] Mithen V, et al. Aboriginal patient and interpreter perspectives on the delivery of culturally safe hospital-based care. Health Promot J Austr. 2021;32(S1):155–65. 32888378 10.1002/hpja.415

[15] Skinner T, et al. Comparative validation of self-report measures of negative attitudes towards Aboriginal Australians and Torres Strait Islanders. Rural Remote Health. 2013;13:1959. 10.22605/RRH1959. 23565853

[16] Silverman J, Kurtz SM, Draper J. Skills for communicating with patients. Third edition / New and updated edition. ed. London; Radcliffe Publishing; 2013. 10.1201/9781910227268.

[17] Ralph AP, et al. Low uptake of Aboriginal interpreters in healthcare: exploration of current use in Australia’s Northern Territory. BMC Health Serv Res. 2017;17(1):733. 29141623 10.1186/s12913-017-2689-y PMC5688693

[18] Álvaro Aranda C, Gutiérrez RL, Li S. Towards a collaborative structure of interpreter-mediated medical consultations: complementing functions between healthcare interpreters and providers. Social Sci Med. 2021;269:113529. 10.1016/j.socscimed.2020.113529 33309440

[19] Lovell J, Clark L. Implementing interventions to improve health communication equity for first nations people: guidance from a rapid realist review. J Health Commun. 2022;27(8):555–62. 36217757 10.1080/10810730.2022.2134523

[20] Lancet T. The soft science of medicine. The Lancet. 2004;363(9417):1247. 10.1016/S0140-6736(04)16027-3 15094264

[21] Wilson D, Barton P. Indigenous hospital experiences: a New Zealand case study. J Clin Nurs. 2012;21(15–16):2316–26. 22642700 10.1111/j.1365-2702.2011.04042.x

[22] Garvey G, et al. The fabric of Aboriginal and Torres Strait Islander wellbeing: a conceptual model. Int J Environ Res Public Health. 2021;18:7745. 34360037 10.3390/ijerph18157745 PMC8345714

[23] Butler TL, et al. Aboriginal and Torres Strait Islander people’s domains of wellbeing: a comprehensive literature review. Soc Sci Med. 2019;233:138–57. 31200269 10.1016/j.socscimed.2019.06.004

[24] Delgado R. Storytelling for oppositionists and others: a plea for narrative. Mich Law Rev. 1989;87(8):2411–41.

[25] Taylor EV, et al. “We’re very much part of the team here”: a culture of respect for Indigenous health workforce transforms Indigenous health care. PLoS ONE. 2020;15(9):e0239207. 32960933 10.1371/journal.pone.0239207 PMC7508383

[26] Taylor KP, et al. Exploring the impact of an Aboriginal health worker on hospitalised aboriginal experiences: lessons from cardiology. Aust Health Rev. 2009;33(4):549–57. 20166903 10.1071/ah090549

[27] Australian Health Practitioner Regulation Agency. Ahpra and national boards annual report 2022/23. Australia: Melbourne; 2023.

[28] Topp SM, Edelman A, Taylor S. “We are everything to everyone”: a systematic review of factors influencing the accountability relationships of Aboriginal and Torres Strait Islander health workers (AHWs) in the Australian health system. Int J Equity Health. 2018;17(1):67. 29848331 10.1186/s12939-018-0779-z PMC5977558

[29] Taylor PS, Habibis D. Widening the gap: White ignorance, race relations and the consequences for Aboriginal people in Australia. Australian J Social Issues. 2020;55(3):354–71.

[30] Freire P. Pedagogy of the oppressed. 30th Anniversary Edition ed. New York: The Continuum International Publishing Group; 1970

[31] Ramsden IM. Cultural safety and Nursing education in Aotearoa and Te Waipounamu. 2002, Victoria University of Wellington: Wellington, New Zealand. 2002; p. 211.

[32] Grogan J, Hollinsworth D, Carter J. Using videoed stories to convey indigenous ‘voices’ in indigenous studies. Australian J Indigenous Education. 2019;50(1):38–46.

[33] Sjorberg D, McDermott D. The deconstruction exercise: an assessment tool for enhancing critical thinking in cultural safety education. Int J Critical Indigenous Stud. 2016;9(1):28–48.

[34] Wain T, et al. Engaging Australian Aboriginal narratives to challenge attitudes and create empathy in health care: a methodological perspective. BMC Med Educ. 2016;16:156. 27255769 10.1186/s12909-016-0677-2 PMC4890246

[35] Haapanen KA, et al. Stories of self, us, and now: narrative and power for health equity in grassroots community organizing. Front Public Health. 2023;11. 10.3389/fpubh.2023.1144123. 10.3389/fpubh.2023.1144123 PMC10286130 37361159

[36] Geia L, et al. A unified call to action from Australian nursing and midwifery leaders:ensuring that Black lives matter. Contemp Nurse. 2020;56(4):297–308. 32799620 10.1080/10376178.2020.1809107

[37] Baum F, MacDougall C, Smith D. Participatory action research. J Epidemiol Community Health. 2006;60(10):854. 16973531 10.1136/jech.2004.028662 PMC2566051

[38] Ralph AP, et al. Improving outcomes for hospitalised First Nations peoples though greater cultural safety and better communication: the Communicate Study Partnership study protocol. Implement Sci. 2023;18(1):23. 37349837 10.1186/s13012-023-01276-1 PMC10286504

[39] Delgado R, Stefancic J, Harris A. Critical race theory: an introduction. 3rd ed. New York: New York University Press. 2017. 10.18574/9781479851393.

[40] Smith, LT. Decolonizing methodologies: research and indigenous peoples. 2nd ed. Zed Books, distributed in the USA exclusively by Palgrave Macmillan; 2012.

[41] Charmaz K. Constructing grounded theory. 2nd ed. London: Sage; 2014.

[42] Simmons OE, Gregory TA. Grounded action: achieving optimal and sustainable change. Forum Qualitative Sozialforschung / Forum: Qualitative Social Research. 2003;4(3):1–7.

[43] Northern Territory Government. Department of Health Annual Report 2022–2023: Darwin, Australia. 2023. Available: https://health.nt.gov.au/__data/assets/pdf_file/0006/1302738/nt-health-annual-report-2022-23.pdf.

[44] Kerrigan V, et al. “How can I do more?” Cultural awareness training for hospital-based healthcare providers working with high Aboriginal caseload. BMC Med Educ. 2020;20(1):173. 32471490 10.1186/s12909-020-02086-5 PMC7260793

[45] Kerrigan V, et al. “The talking bit of medicine, that’s the most important bit”: doctors and Aboriginal interpreters collaborate to transform culturally competent hospital care. Int J Equity in Health. 2021;20(1):170. 34301261 10.1186/s12939-021-01507-1 PMC8299635

[46] Patton MQ. Qualitative evaluation and research methods. Beverly Hills, Calif.: Sage Publications; 1990. pp. 169–186.

[47] Whyte K. Against crisis epistemology, In: Hokowhitu B, et al., editors. Routledge Handbook of Critical Indigenous Studies. Routledge International Handbooks; 2021. pp. 52–64.

[48] Bernal DD. Using a Chicana feminist epistemology in educational research. Harv Educ Rev. 1998;68(4):555–81.

[49] Gallagher M. Voice audio methods. Qualitative Res: QR. 2020;20(4):449–64.

[50] Bargallie D. Unmasking the racial contract: indigenous voices on racism in the Australian public service. 1st ed. Canberra, ACT: Aboriginal Studies Press; 2020. p. 256.

[51] Kowal E. The politics of the gap: indigenous Australians, liberal multiculturalism, and the end of the self-determination era. Am Anthropol. 2008;110(3):338–48.

[52] Bonilla-Silva E. The structure of racism in color-blind, “post-racial” America. Am Behav Sci (Beverly Hills). 2015;59(11):1358–76.

[53] Bashford A. “Is White Australia possible?” Race, colonialism and tropical medicine. Ethn Racial Stud. 2000;23(2):248–71.

[54] Australian Commission on Safety and Quality in Health Care. National safety and quality health service standards. User guide for Aboriginal and Torres Strait Islander Health. Sydney, NSW. 2017. Available: https://www.safetyandquality.gov.au/sites/default/files/migrated/National-Safety-and-Quality-Health-Service-Standards-User-Guide-for-Aboriginal-and-Torres-Strait-Islander-Health.pdf.

[55] Wicks M, et al. Racial microaggressions and interculturality in remote Central Australian Aboriginal healthcare. Int J Equity Health. 2023;22(1):103. 37231471 10.1186/s12939-023-01897-4 PMC10211281

[56] Jones CP. Levels of racism: A theoretic framework and a gardener’s tale. Am J Public Health. 2000;90(8):1212–5. 10936998 10.2105/ajph.90.8.1212 PMC1446334

[57] Elias A, Mansouri F, Paradies Y. Racism in Australia today. Gateway East, Singapore: Palgrave Macmillan; 2021.

[58] Essed P. Understanding Everyday Racism: an interdisciplinary theory. 1st ed. vol. 2, SAGE Publications, Incorporated, 1991, pp. x–x. 10.4135/9781483345239.

[59] Gatwiri K, Rotumah D, Rix E. BlackLivesMatter in healthcare: racism and implications for health inequity among Aboriginal and Torres Strait Islander peoples in Australia. Int J Environ Res Public Health. 2021;18(9):4399. 33919080 10.3390/ijerph18094399 PMC8122304

[60] Bargallie D, Fernando N, Lentin A. Breaking the racial silence: Putting racial literacy to work in Australia. Ethnic and Racial Studies. 2024;47(8):1532–51. 10.1080/01419870.2023.2206470.

[61] Wylie L, McConkey S. Insiders’ insight: discrimination against indigenous peoples through the eyes of health care professionals. J Racial Ethn Health Disparities. 2019;6(1):37–45. 29736617 10.1007/s40615-018-0495-9 PMC6347580

[62] Abbas J, et al. The role of social media in the advent of covid-19 pandemic: crisis management, mental health challenges and implications. Risk Manag Healthcare Policy. 2021;14:1917–32. 10.2147/RMHP.S284313 PMC8126999 34012304

[63] Paradies Y, Truong M, Priest N. A systematic review of the extent and measurement of healthcare provider racism. J Gen Intern Med. 2014;29(2):364–87. 24002624 10.1007/s11606-013-2583-1 PMC3912280

[64] Horrill T, et al. Understanding access to healthcare among Indigenous peoples: a comparative analysis of biomedical and postcolonial perspectives. Nursing Inquiry. 2018;25(3):e12237. 29575412 10.1111/nin.12237 PMC6055798

[65] Hall ET. Beyond culture. Anchor books. Garden City, N.Y: Anchor Press; 1977.

[66] Moodie N. Decolonizing race theory: place, survivance & sovereignty, In: Vass G, et al., editors. The relationality of race in education research. Routledge: New York; 2018 p. 182. 10.4324/9781315144146.

[67] Nakata M. The cultural interface. Aust J Indigenous Education. 2007;36(S1):7–14.

[68] Lowell A, Maypilama EL, Gundjarranbuy R. Finding a pathway and making it strong: Learning from Yolŋu about meaningful health education in a remote Indigenous Australian context. Health Promot J Austr. 2021;32(S1):166–78. 32808441 10.1002/hpja.405

[69] McGrath S, et al. The Voice to Parliament “creates an opportunity to codesign a healthy future”. 4 September 2023: MJA Insight+. Available: https://insightplus.mja.com.au/2023/33/the-voice-to-parliament-creates-an-opportunity-to-codesign-a-healthy-future/.

[70] Armstrong E, et al. Räl-manapanmirr ga dhä-manapanmirr - Collaborating and connecting: Creating an educational process and multimedia resources to facilitate intercultural communication. Int J Speech Lang Pathol. 2022;24(5):533–46. 10.1080/17549507.2022.2070670. 35633090 10.1080/17549507.2022.2070670

[71] Herdman RM, Rafferty C. Gurruṯu: a system of communicating and collaborating in intercultural spaces. In First Nations Health Communication Symposium. Darwin, Australia: Menzies School of Health Research; 2023.

[72] Peoples WA, Fleming PJ, Creary MS. Working toward health equity requires antiracist teaching. Am J Preventive Med. 2023;64(4):604–8. 10.1016/j.amepre.2022.10.023 36690544

[73] Walmsley AJ, Baker DR, Lowell A. Bakmaranhawuy - the broken connection. Perspectives on asking and answering questions with Yolŋu patients in healthcare contexts. Rural Remote Health. 2022;22(1):6959. 35044785 10.22605/RRH6959

[74] Armstrong E, et al. Nhaltjan dhu ḻarrum ga dharaŋan dhuḏi-dhäwuw ŋunhi limurr dhu gumurrbunanhamirr ga waŋanhamirr, Yolŋu ga Balanda: how we come together to explore and understand the deeper story of intercultural communication in a Yolŋu (First Nations Australian) community. AlterNative: Int J Indigenous Peoples. 2023;19(2):334–44. 10.1177/11771801231169337.

[75] Kurtz SM, Draper J, Silverman J. Teaching and learning communication skills in medicine. 2nd ed. Oxford: Radcliffe Publishing; 2005.

[76] Kerrigan V, et al. Evaluating the impact of ‘Ask the Specialist Plus’: a training program for improving cultural safety and communication in hospital-based healthcare. BMC Health Serv Res. 2024;24(1):1–13. 38254093 10.1186/s12913-024-10565-4 PMC10804863

[77] Oster RT, et al. Cultural continuity, traditional Indigenous language, and diabetes in Alberta First Nations: a mixed methods study. Int J Equity in Health. 2014;13(1):92. 25326227 10.1186/s12939-014-0092-4 PMC4210509

[78] Taylor K. Health care and Indigenous Australians: cultural safety in practice. 2nd ed. South Yarra, VIC: Palgrave Macmillan; 2014.

[79] Kerrigan V, et al. Evaluation of “Ask the Specialist”: a cultural education podcast to inspire improved healthcare for Aboriginal peoples in Northern Australia. Health Sociol Rev. 2022;31(2):139–57. 35373706 10.1080/14461242.2022.2055484

[80] Norman R. Report to the Department of Health and Families NT on Governance of complaint handling and implementation of open disclosure at Royal Darwin Hospital. 30/1/2009 NT Department of Health and Families: Darwin, NT; 2009.

[81] Jones B, Heslop D, Harrison R. Seldom heard voices: a meta-narrative systematic review of Aboriginal and Torres Strait Islander peoples healthcare experiences. Int J Equity Health. 2020;19(1):222. 33317556 10.1186/s12939-020-01334-w PMC7734845

[82] Goodyear L, Barela E. Qualitative inquiry in evaluation: from theory to practice. 1st ed. vol. 29, Wiley, 2014.

