# Supplementary material for: “If They Help Us, We Can Help Them”: First Nations Peoples Identify Intercultural Health Communication Problems and Solutions in Hospital in Northern Australia
Source: J Racial Ethn Health Disparities. 2024 Sep 30;12(6):3601–12. doi: 10.1007/s40615-024-02160-4 (PMC12644241; doi:10.1007/s40615-024-02160-4)
Supplement: Supplementary file 1 — Supplementary file1 (DOC 147 KB) [file 40615_2024_2160_MOESM1_ESM.doc]

**Title**: “*If they help us, we can help them*”: First Nations peoples identify intercultural health communication problems and solutions in hospital in northern Australia

**Journal**: Racial and Ethnic Health Disparities

**Authors:** Vicki Kerrigan, Stuart Yiwarr McGrath, Rachel Dikul Baker, Jeanette Burrunali, Anna P Ralph, Rarrtjiwuy Melanie Herdman, Tiana Alley and Emily Armstrong

**Corresponding author:** Vicki Kerrigan – vicki.kerrigan@menzies.edu.au

Menzies School of Health Research, Charles Darwin University, PO Box 41096, Casuarina, NT 0811, Australia. ORCID ID: 0000-0001-6863-1528

**PATIENT EXPERIENCE INTERVIEW GUIDE**

THE COMMUNICATE STUDY: Partnership across the Top End to improve Aboriginal patients’ experience and outcomes of healthcare

Interviews will be semi-structured to allow for issues of relevance to participants to emerge. The interviewer will follow the conversational lead set by the participant. Key discussion points will include (but not be limited to):

**Introduction/background**

1. Tell me a bit about you and your family:
   1. What’s your name?
   2. Where are you from? Who’s your family (ie. mob, Nation, clan)?
   3. Language?
   4. Have the hospital staff been asking you questions like this too? (Do the staff know who you are? Do they know where you’re from? Have you been talking to anyone about your family?)
2. What do you do when you’re not at hospital? (explore family role, community role, professional role etc)

**Your health, wellbeing and hospital experience**

1. Can you tell me why you came to hospital?
   1. How long have you been in hospital?
   2. Is this the first time you have been to hospital?
   3. If no, can you tell me about the other times you have been to hospital? Why did you come to hospital? Why did you come back to hospital this time?
2. How do you feel about being in hospital? (physical, mental, spiritual, holistic wellbeing)
3. When you talk to your family about being in hospital, what do you tell them about?
4. What are your thoughts about your own health and wellbeing?
5. What would make you feel well/ better?
6. Do you have plans for your own healing? (e.g. patients might talk about alternative treatments or other activities to build strength and wellbeing)
7. How do the staff treat you here? (Have you experienced feeling welcome? Have you experienced racism?)
8. Do you feel respected?
9. What do the staff do or say to make you feel this way?

**Cultural safety in the hospital** (explain the concept of cultural safety to the patient)

7. Do you feel safe and comfortable here in the hospital? (emotionally, mentally, physically) 8. Do you feel like people are helping you? Who has helped you here in hospital? How do they help you? How do they make you feel?

- 1. Staff - Doctors or nurses or AHP’s or Aboriginal Liaison Officers or interpreters or the bus driver or cleaner? (ask about each of these roles because patients interact with many professionals)
  2. Other patients and family?
  3. How comfortable to do feel sharing your worries with these people? (Are there some things you can only share with someone you know? Are there things you want to keep confidential?)

1. Has an Aboriginal Liaison Officer (ALO) helped you?
   1. What did they help you with?
   2. How did it make you feel to be able to speak to an ALO?
2. Have you had visitors while you’ve been here?
   1. If yes, who has come to see you? (explore reasons for people being able to visit/not visit)
   2. How do your visitors support you?
   3. How does it feel to have visits from people you know?
3. Would you like to share any worries you have while you are in hospital? (e.g. feeling frightened; feeling unsure of healthcare; being far from family; other responsibilities)
   1. Have you been able to share your worries with the staff here? (If not, why not?)
   2. Have you been able to ask for help? (Probe for reasons about why they have/have not been able to ask for help)
   3. Do you know who to talk to or what to do if you weren’t happy with your hospital care?

d. What would help fix the worries? (What could be changed? What could staff do differently? What could you do?)

1. Do you ever feel like hospital is a racist place? (If yes, what makes you feel that way?)
   1. What could the hospital staff do to make patients feel more respected here?
2. No one wants to be in hospital, but if you have to be in hospital, what would make the hospital feel safer and comfortable for you and your family?
3. How do these spaces and buildings feel to you?
4. Do you think about leaving hospital because you feel uncomfortable or not safe?
5. Have you ever left hospital without telling the doctors or nurses that you had to leave? (explore reasons why)
6. If you were going to train up the staff in how to work with Aboriginal patients, what would you teach them?

**Communicating with the hospital staff**

1. Do you understand what is making you sick? Do you understand what might make you get better?
2. How did you find out about your health condition? (e.g. who told you, how did they explain it?)
3. Do people in this hospital listen to you? Do they understand you?
4. Do you feel you have some control over what is happening to you in hospital?
   1. Have you filled out any consent forms? Did you understand them? What helped/ would help you understand?
5. If you are confused, what do you do? Do you ask questions?
   1. Do you sometimes say ‘yes’ even if you don’t understand or are confused? (If so, why do you agree with what the health professional says?)

**Aboriginal languages (if patient speaks an Aboriginal language as first language)**

1. Have you had access to an interpreter?
2. Who was it? And are you related to the interpreter?
3. What did they help you with? What did they help the staff with?
4. How did you feel when you can communicate in your first language?
5. Why is it important to speak in your first language?

**Is there anything else you want to share with the researchers?**
